# A multivalent vaccine candidate targeting enterotoxigenic *Escherichia coli* fimbriae for broadly protecting against porcine post-weaning diarrhea

**DOI:** 10.1186/s13567-020-00818-5

**Published:** 2020-07-23

**Authors:** Qiangde Duan, Shengmei Pang, Wenwen Wu, Boyu Jiang, Weiping Zhang, Siguo Liu, Xiaojun Wang, Zhiming Pan, Guoqiang Zhu

**Affiliations:** 1 College of Veterinary Medicine (Institute of Comparative Medicine), Yangzhou University, 12 East Wenhui Road, Yangzhou, 225009 China; 2 Jiangsu Co-Innovation Center for Prevention and Control of Important Animal Infectious Diseases and Zoonoses, Joint International Research Laboratory of Agriculture and Agri-Product Safety of Ministry of Education of China, Yangzhou, China; 3 Department of Pathobiology, University of Illinois at Urbana-Champaign, Urbana, IL 61802 USA; 4 Harbin Veterinary Research Institute, Chinese Academy of Agricultural Sciences, Harbin, China

**Keywords:** ETEC, PWD, Fimbriae, MEFA, Vaccine

## Abstract

Fimbriae-mediated initial adherence is the initial and critical step required for enterotoxigenic *Escherichia coli* (ETEC) infection. Therefore, vaccine candidates have been developed that target these fimbriae and induce specific anti-fimbriae antibodies to block initial ETEC attachment. While this vaccine effectively protects against ETEC-associated post-weaning diarrhea (PWD), developing a broadly effective vaccine against initial ETEC attachment remains a challenging problem, owing to the immunological heterogeneity among these antigens. Here, we applied multi-epitope fusion antigen (MEFA) technology to construct a FaeG–FedF–FanC–FasA–Fim41a MEFA using the adhesive subunits of predominant fimbriae K88 and F18 as the backbone, which also integrated epitopes from adhesive subunits of the rare fimbriae K99, 987P, and F41; we then generated a MEFA computational model and tested the immunogenicity of this MEFA protein in immunized mice. We next evaluated the potential of the fimbriae-targeted MEFA as a vaccine candidate to effectively prevent PWD using in vitro assessment of its anti-fimbriae, antibody-directed inhibition of bacterial adherence. Computational modeling showed that all relevant epitopes were exposed on the MEFA surface and mice subcutaneously immunized with the MEFA protein developed IgG antibodies to all five fimbriae. Moreover, anti-fimbriae antibodies induced by the MEFA protein significantly inhibited the adhesion of K88+, F18+, K99+, 987P+, and F41+ ETEC strains to piglet small intestinal IPEC-1 and IPEC-J2 cell lines. Taken together, these results indicate that FaeG–FedF–FanC–FasA–Fim41a MEFA protein induced specific anti-fimbriae neutralizing antibodies against the five targeted fimbriae. Critically, these results show the potential of fimbriae-targeted MEFA and indicate their promise as a broad, effective vaccine against PWD.

## Introduction

Enterotoxigenic *Escherichia coli* (ETEC) characteristically produces two types of virulence factors: fimbriae and enterotoxins. ETEC is recognized as a major bacterial cause of diarrhea in young children in lower-income countries as well as in international travelers; it is also a cause of diarrhea in neonatal and post-weaning piglets [1–3]. Fimbriae and enterotoxins are the two prominent virulence determinants that contribute to ETEC-associated diarrhea. Fimbriae promote the pathogen’s initial binding to specific receptors on the target host cell and subsequent colonization of the host’s intestinal epithelial cells. Molecular epidemiological studies, from cases of calibacillosis in pigs, have indicated that post-weaning diarrhea (PWD) is primarily caused by ETEC strains expressing K88 and F18 fimbriae, with the K88ac variant of the K88 fimbria being the most prevalent [4, 5]. Although K99, 987P, and F41 fimbriae are usually associated with neonatal diarrhea, they are occasionally found in PWD infections [4, 6, 7]. Despite the difference in the antigenic classification of K88 serological variants (e.g., K88ab, ac, ad), the major structural subunit—FaeG—has been recognized as the common adhesive subunit to all variants [8]. When it comes to the F18 fimbria, two antigenic variants—F18ab and F18ac—have been identified. Of these, the F18ac variant has been frequently related to PWD, while the F18ab variant has been more associated with porcine edema disease (ED) [9]. The adhesive subunit of the F18 fimbria is its minor subunit—FedF—and this subunit is highly conserved between the two antigenic variants [10]. Once colonization of the small intestinal epithelial cells occurs, ETEC bacteria secrete two classes of enterotoxins: heat-labile enterotoxin (LT) and heat-stable enterotoxin (ST). Collectively, these toxins stimulate the intestinal lining to secrete excessive fluid, thus causing diarrhea. The initial subsequent attachment between the adhesive subunit of the fimbriae and special receptors on host cells facilitates efficient delivery of enterotoxins and progression of infection pathogenesis, both of which play important roles in ETEC infections.

Newly weaned piglets are very susceptible to PWD, which remains a major disease in the swine industry and accounts for substantial global economic losses [1]. PWD causes a variety of symptoms in newly weaned piglets, including weight loss, profuse watery diarrhea, and even acute death, and remains a major challenge for the industry. Various prevention strategies have been tried to control and prevent PWD, including treatment with antibiotics [11], passive administration with specific antibodies [12], dietary supplementation [13], genetic breeding programs to generate ETEC-resistant stock [14] and vaccination [15]. However, none have been effective in protecting against PWD. Currently, treatment with antibiotics during the first 2 weeks post-weaning can relieve ETEC-associated diarrhea and other clinical symptoms. However, inappropriate and excessive use of antibiotics has led to animal health problems such as induced antimicrobial resistance in bacteria, which can cause diseases in animals [16]. Oral administration of specific anti-fimbriae antibodies to pregnant sows can provide a small amount of protection during feeding, but is both expensive and labor intensive [17]. Regarding genetic breeding strategies, it remains difficult to not only identify specific genetic markers, but also apply them to screen ETEC resistant or susceptible pigs. Given this persistent problem, a strong need for alternative strategies for the prevention and treatment of PWD remains. Vaccination is considered an ideal and effective approach to better control and protect against ETEC-caused PWD. One of ETEC’s major virulent determining fimbria has received significant attention owing to its essential role in ETEC initial adherence and good immunogenicity [2]. In the past decades, vaccine development efforts have focused primarily on using ETEC fimbriae as antigens to induce production of anti-fimbriae antibodies. These antibodies then block the initial adherence of ETEC. Pigs have been vaccinated with cocktail products that contain the whole, dead ETEC bacteria expressing various fimbriae, avirulent strains expressing ETEC fimbriae, purified fimbriae, or fimbriae adhesive subunit. In all cases, these vaccines were able to resist PWD caused by ETEC expressing the corresponding fimbriae [18, 19]. Recently, a commercialized, live, and non-pathogenic vaccine for F4+ and F18+ ETEC was shown to protect newly weaned piglets who had been challenged with both F4+ and F18+ ETEC. Impressively, this protection was achieved with only one, oral dose [20]. Vaccines administrated via the oral route will rapidly trigger mucosal immunity and induce production of antigen-specific sIgA antibodies, which will block the colonization of the pathogenic bacteria and protect against ETEC-caused PWD. However, these vaccines are unable to provide cross-protection against PWD caused by ETEC expressing different fimbriae, owing to the heterogeneity of these antigens. Though the vast majority of ETEC causing PWD mostly express K88 or F18 fimbriae, ETEC expressing K99, 987P and F41 fimbriae are usually found in ETEC caused neonatal diarrhea and also occasionally in PWD infection. Luppi et al. [6] reported that the prevalence rate of K99, 987P and F41 fimbriae among ETEC isolated from cases of PWD was 0.6%, 0.6% and 0.3%, respectively in Europe. In China, F5 (12%), F6 (7.2%) and F41 (6%) were also frequently detected, suggesting that these fimbriae were closely related to ETEC isolated from piglets with PWD [7]. Therefore, a vaccine that encompasses all five ETEC fimbriae would be more effective in providing holistic protection against ETEC-driven PWD.

In this study, we applied an epitope- and structure-based vaccinology platform to develop a fimbriae-targeted, multi-epitope fusion antigen (MEFA) protein. The MEFA protein carried all five porcine ETEC fimbriae epitopes and used both the FaeG subunit of the K88 fimbria and the FedF subunit of the F18 fimbria as its backbone. Immunogenicity of the MEFA fimbriae was tested using specific anti-fimbriae antibodies obtained from immunized mouse serum. Furthermore, the level of in vitro inhibition of ETEC adherence to piglet small intestinal epithelial cells by MEFA-induced anti-fimbriae antibodies was also determined. Finally, we also evaluated the potential for using these fimbriae MEFA in establishing an effective, broad-spectrum vaccine to prevent PWD.

## Materials and methods

### Bacterial strains, plasmids, and cell line culture conditions

The bacterial strains and plasmids used in this study are listed in Table 1. Wild-type porcine ETEC strains expressing K88, K99, 987P, F18, or F41 fimbriae (China Institute of Veterinary Drugs Control, CIVDC) were used as templates for PCR amplification of fimbriae adhesive subunit genes and also used in in vitro porcine ETEC antibody adherence inhibition assays. Expression vector pET28α+ (Novagen, Madison, WI) and *E. coli* strains DH5α and BL21 (DE3) were used for gene cloning and recombinant protein expression. Porcine intestinal epithelial cell line IPEC-1 and neonatal jejunal epithelial cell line IPEC-J2 were cultured in Dulbecco minimal Eagle medium (DMEM) supplemented with 10% heat-inactivated fetal bovine serum (FBS) (Gibco) and maintained in an atmosphere of 5% CO_2_ at 37 °C.

**Table 1:** **Strains and plasmids used in this study.**

Strains/plasmids	Relevant properties	Source
BL21	F^−^*omp*T *hsd*S(r_B_^−^m_B_^−^) *gal dcm*	Novagen
C83901	wild-type O8: H19:K88ac LT/STa/STb	CIVDC
2134P	wild-type O157:H19 F18ac/4p-/STa/STb	Casey et al. [31]
1592	Wild-type 987P+ ETEC	CIVDC
1593	Wild-type K99+ ETEC, STa	CIVDC
1594	Wild-type F41+ ETEC	CIVDC
1901	FaeG–F41–FanC–FasA MEFA recombinant strain	This study
1902	FedF–FasA–F41–FanC MEFA recombinant strain	This study
1903	FaeG subunit (K88 fimbriae) recombinant strain	This study
1904	FanC subunit (K99 fimbriae) recombinant strain	This study
1905	FasA subunit (987P fimbriae) recombinant strain	This study
1906	FedF subunit (F18 fimbriae) recombinant strain	This study
1907	Fim41a subunit (F41 fimbriae) recombinant strain	This study
Plasmid		
pET28α+		Novagen
p1908	FaeG–F41–FanC–FasA MEFA in pET28α+	This study
p1909	FedF–FasA–F41–FanC MEFA in pET28α+	This study
p1910	FaeG–FedF–F41–FanC–FasA MEFA in pET28α+	This study

### Construction of FaeG–FedF–F41–FanC–FasA MEFA and adhesive subunit genes

The FaeG subunit of the K88 fimbria and the FedF subunit of the F18 fimbria were both used as the backbone for embedding nucleotide segments coding for the neutralized epitopes of the FanC subunit of the K99 fimbria, the FasA subunit of the 987P fimbria, and the Fim41a subunit of the F41 fimbria. These subunits were all identified using a web-based B-cell epitope software, as previously described [21]. Three, less antigenic epitopes in either the FaeG or FedF subunit were substituted with nucleotide fragments coding for the most antigenic epitopes of the FasA, FanC, or Fim41a subunits. The resulting *faeg*–*fim41a*–*fanc*–*fasa* and *fedf*–*fasa*–*fim41a*–*fanc* chimeric genes were synthesized by Takara Biotechnology Co. Ltd. (Dalin, China) after optimizing the epitope substitution and MEFA protein structure using both a tree-dimension protein modeling program and a PyMOL molecular graphics system. These two chimeric genes were then fused to the *faeg*–*fedf*–*fanc*–*fasa*–*fim41a* chimeric gene by applying splicing overlap extension (SOE) PCR with P1 and P2 primers (Table 2). The PCR primers used to amplify the fimbriae adhesive subunit genes *faeg*, *fanc*, *fasa*, *fedf,* and *fim41a* are listed in Table 2. The chimeric and fimbriae subunit gene products were cloned into a restriction enzyme digested pET28α+ vector.

**Table 2:** **Primers used in this study.**

Primer	Sequences (5′-3′)
MEFA-F1	CGCGGATCCATGAAAAAAACCCTGATCGCTCTGGC
MEFA-F2	ATCACCTACTACGGACCCGGACCTGGTCGTCTGAAATAC
MEFA-R1	GAGCGTCGACTTACTGGATTTCGAA AACGATCGGAAC
MEFA-R2	GTATTTCAGACGACCAGGTCCGGGTCCGTAGTAGGTGAT
FasA-F	CGCGGATCCATGAGAATGAAAAAATCCGC
FasA-R	CAGCGTCGACTTACGGTGTACCTGCTGAA
FanC-F	CGCGGATCCATGAAAAAAACACTGCTAGC
FanC-R	CAGCGTCGACTTACATATAAGTGACTAAG
FedF-F	CTAGCTAGCATGCGTTTAAAATATATCTTG
FedF-R	CAGCGTCGACTTACTGTATCTCGAAAAC
FaeG-F	ATTCGGGATCCATGAAA AAGAC
FaeG-R	CAGCGTCGACTTAGTAATAAGT
Fim41a-F	CGCGGATCCATGAAAAAGACTCTGATTGC
Fim41a-R	AACGAGCTCTTAACTATAAATAACGGTG

Restriction sites, BamHI or NheI in forward primers, and SalI or SacI in reverse primers, were underlined.

### Expression and purification of recombinant proteins

Recombinant ETEC fimbriae-based MEFA and fimbriae subunit proteins were expressed and purified as previously described [21]. Briefly, a single recombinant *E. coli* colony was subcultured overnight in 2×YT medium (2× Yeast Extract Tryptone) containing kanamycin at a final concentration of 30 μg/mL. Bacteria were then allowed to grow at 37 °C with vigorous shaking at 220 rpm. Then, 2 mL of this overnight subculture were transferred to 200 mL of fresh 2×YT medium supplemented with 30 μg/mL kanamycin. When the optical density of the bacterial culture reached 0.6 at 600 nm (OD_600_), bacterial culture was induced by 1 mM isopropyl-β-d-1-thiogalactoside (IPTG) for an additional 4 h. Bacteria pellets were then harvested by centrifugation at 12 000 rpm for 15 min. Total recombinant protein was extracted with a bacterial protein extraction reagent (B-PER) (Thermo Scientific, Rochester, NY). Extracted recombinant 6× His tagged inclusion body proteins were purified from the total protein extract using protino^○R^Ni-TED 2000 packed columns (MACHEREY-NAGEL, Germany) according to the manufacturer’s instructions. Purified MEFA protein was examined via 12% sodium dodecyl sulfate polyacrylamide gel electrophoresis (SDS-PAGE) with Coomassie blue staining and subsequent western blot with anti-FaeG monoclonal antibody (1:1000), anti-FedF serum (1:4000), anti-FanC serum (1:4000), anti-FasA serum (1:4000), and anti-Fim41a serum (1:4000).

### Subcutaneous immunization of mice with fimbrial MEFA

Animal experiments were approved by and performed at the Animal Experiment Center of Yangzhou University (Yangzhou, China). All animal experiments followed the National Institute of Health guidelines for the ethical use of animals in China.

A total of 45, 8-week-old female BALB/c mice (Laboratory Animal Center of Yangzhou University, Jiangsu, China) were divided into three groups, with 15 mice per group. In group 1, mice were SC co-immunized with 50 µg of the FaeG–Fim41a–FanC–FasA MEFA protein combined with 50 µg FedF–FasA–Fim41a–FanC. In group 2, mice were SC immunized with only 100 µg of the FaeG–FedF–FanC–FasA–Fim41a MEFA protein. An equal volume of Freund complete adjuvant (Sigma, USA) was used as the adjuvant with the primary immunization. Each mouse received two booster injections at the same dose as the primary immunization, but with Freund incomplete adjuvant (Sigma, USA). These boosters occurred at 2-week intervals. In group 3, mice were injected only with 200 µL of sterile phosphate buffer saline (PBS) and were used as the control group. All mice were euthanized 2 weeks after the second booster. Blood samples were collected from each mouse both before immunization and at the time of euthanasia.

### Mouse serum anti-fimbriae specific IgG antibody titration

Serum samples from each immunized or control mouse were examined for antibodies specific to FaeG, FedF, FanC, FasA, and Fim41a fimbriae subunits. This analysis was conducted using an enzyme-linked immunosorbent assay (ELISA) as previously described [22]. Briefly, 500 ng purified FaeG, FedF, FanC, FasA, or Fim41a recombinant protein was coated onto each well of a 96-well plate (Corning, USA) and used as the coating antigens. Non-coated spots were blocked via incubation with 10% non-fat milk at 37°C for 1 h, after which the plate was washed with PBST (PBS with 0.05% Tween 20). Mouse serum samples were diluted twofolds from 1:400 to 1:25,600 and examined in the aforementioned ELISA assay. Horseradish peroxidase (HRP)-conjugated goat anti-mouse IgG (1:5000; Sigma, USA) was used as the secondary antibody. The OD_650_ value for each well was measured after exposure to 3, 3′, 5, 5′-tetramethylbenzidine (TMB) HRP color development solution (Beyotime, China) and converted into antibody titers in log_10_ form. All experiments were conducted in triplicate.

### Mouse serum antibody inhibition against adherence of K88+, F18+, K99+, 987P+, and F41+ ETEC strains to porcine intestinal epithelial cells

Porcine small intestinal epithelial cell lines IPEC-1 and IPEC-J2 as well as wild-type ETEC strains expressing K88, F18, K99, 987P, and F41 fimbriae were used to perform in vitro antibody adherence inhibition assessment as previously described [21]. Briefly, wild-type ETEC bacteria in the logarithmic phase were harvested and resuspended in sterile PBS. Bacterial suspensions (MOI of 5 bacteria per cell) were first co-incubated with 30 µL of mouse serum from each group, and then placed on a shaker with gentle agitation for 30 min at room temperature. Each mouse serum/bacteria mixture (final volume of 600 μL) was added to each well of a 24-well tissue culture plate, which contained a confluent monolayer of either IPEC-1 or IPEC-J2 cells. Cells were then incubated in a CO_2_ incubator (5% CO_2_) at 37 °C for 1 h, after which they were rinsed with sterile PBS to remove any non-adherent bacteria. Cells were then dislodged with 0.5% Triton X-100 (Solarbio life Science, China). Dislodged cells containing the adherent ETEC bacteria were serially diluted using PBS and plated on LB agar plates. The number of bacteria colonies (CFU) on LB agar plates were counted after overnight growth in a 37 °C incubator.

### Statistical analysis

Mouse serum IgG antibody titers are presented in log_10_; in vitro mouse serum antibody adherence inhibition assay was analyzed using GraphPad Prism version 6.0 (GraphPad Software, USA). Student *t*-test was used to compare data (means ± standard deviations) between different treatment groups. Differences were considered statistically significant at p < 0.05.

## Results

### Fimbriae-targeted MEFA carried the epitopes of the five fimbriae subunits of porcine ETEC

**Figure 1:**
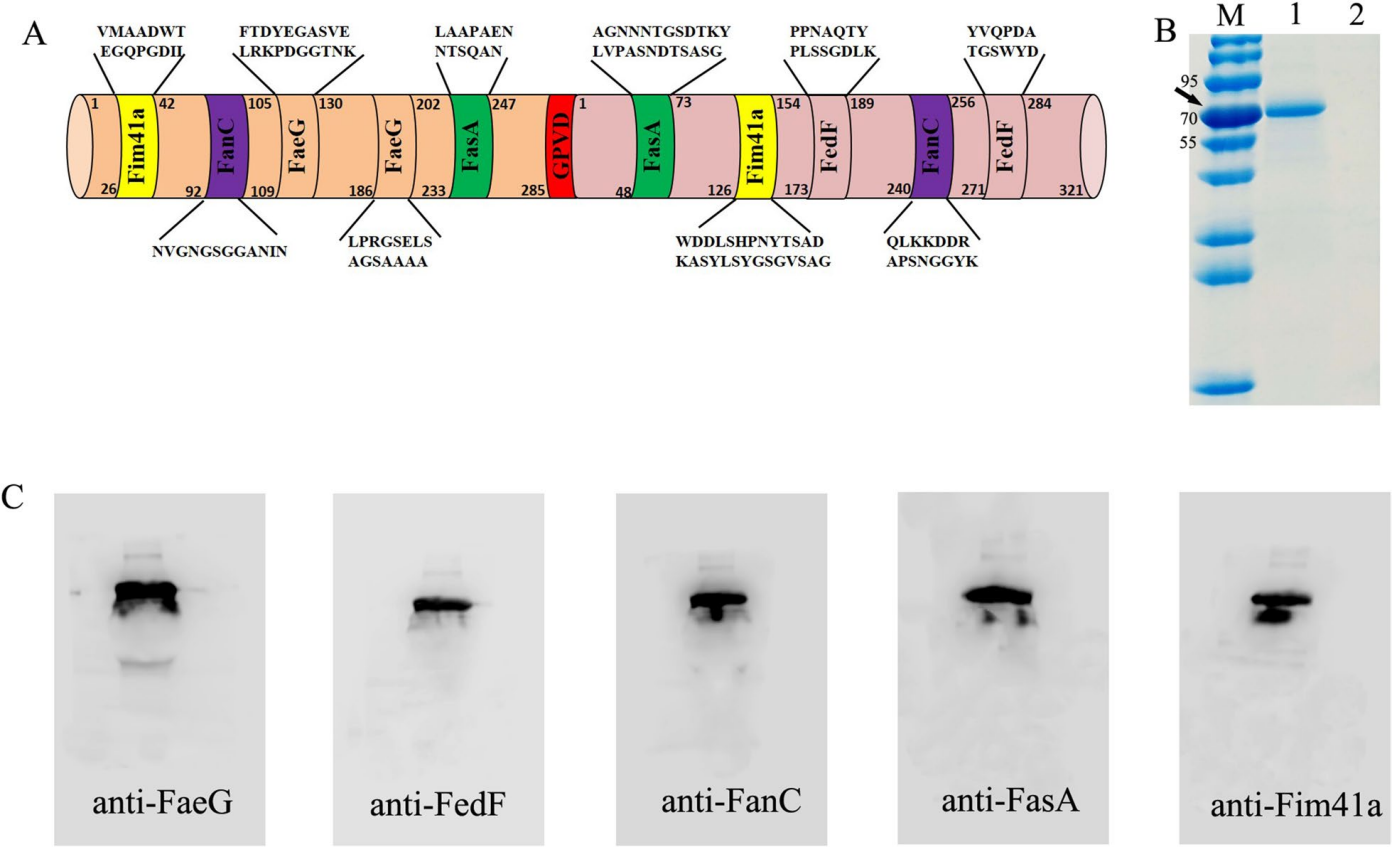
**Construction and detection of FaeG–FedF–FanC–FasA–Fim41a fimbriae MEFA protein. A** Schematic illustration of the constructed ETEC FaeG–FedF–FanC–FasA–Fim41a fimbriae MEFA protein. The K88 fimbriae major structural subunit (FaeG) and the F18 fimbriae minor subunit (FedF) were used as the backbone. Each of these backbone components had nucleotides coding for three surface-exposed—but less-antigenic—epitopes substituted for by nucleotides coding for most antigenic epitopes predicted from K99, 987P, and F41 fimbriae. This allowed for the final construction of the ETEC fimbriae MEFA protein. **B** Detection of purified FaeG–FedF–FanC–FasA–Fim41a MEFA protein by 12% SDS-PAGE and Coomassie blue staining. **C** The expressed FaeG–FedF–FanC–FasA–Fim41a MEFA protein was detected using anti-FaeG monoclonal antibody (1:1000), anti-FedF serum (1:4000), anti-FanC serum (1:4000), anti-FasA serum (1:4000), and anti-Fim41a serum (1:4000) along with an HRP-conjugated goat anti-mouse IgG (1:10,000).

In silico predictions for each fimbriae’s antigens indicate two epitopes (‘NVGNGSGGANIN’ and ‘QLKKDDRAPSNGGYK’) from the K99 fimbria major subunit FanC, two epitopes (‘LAAPAENNTSQAN’ and ‘AGNNNTGSDTKYLV PASNDTSASG’) from the 987P fimbria major subunit FasA, and two epitopes (‘VMAADWTEGQ PGDII’ and ‘WDDLSHPNYTSADKASYLSYGSGVSAG’) from the F41 fimbria major subunit Fim41a. Epitopes ‘FTDYEGASVELRKPDGGTNK’ and ‘LPRGSELSAGSAAAA’ were retained in the FaeG backbone, while epitopes ‘PPNAQTYPLSSGDLK’ and ‘YVQPDATGSW YD’ were retained in the FedF backbone. The epitopes of FanC, FasA, and Fim41a were used to substitute for three less antigenic epitopes of the FaeG or FedF backbone, and to construct either the *faeg*–*fim41a*–*fanc*–*fasa* or *fedf*–*fasa*–*fim41a*–*fanc* chimeric genes. Furthermore, the *faeg*–*fim41a*–*fanc*–*fasa* and *fedf*–*fasa*–*fim41a*–*fanc* genes were fused to a *faeg*–*fedf*–*fanc*–*fasa*–*fim41a* fimbriae MEFA gene using the SOE-PCR method (Figure 1A). The FaeG–FedF–FanC–FasA–Fim41a MEFA protein was then expressed, extracted and purified.

SDS-PAGE in conjunction with Coomassie blue staining revealed a protein with a molecular mass of approximately 70 kDa, which was the expected size of the fimbriae MEFA protein (Figure 1B). The fimbriae MEFA protein was recognized by an anti-FaeG monoclonal antibody as well as anti-FedF, anti-FanC, anti-FasA, and anti-Fim41a antisera (Figure 1C).

### All five fimbriae epitopes were displayed on the fimbriae MEFA protein surface

**Figure 2:**
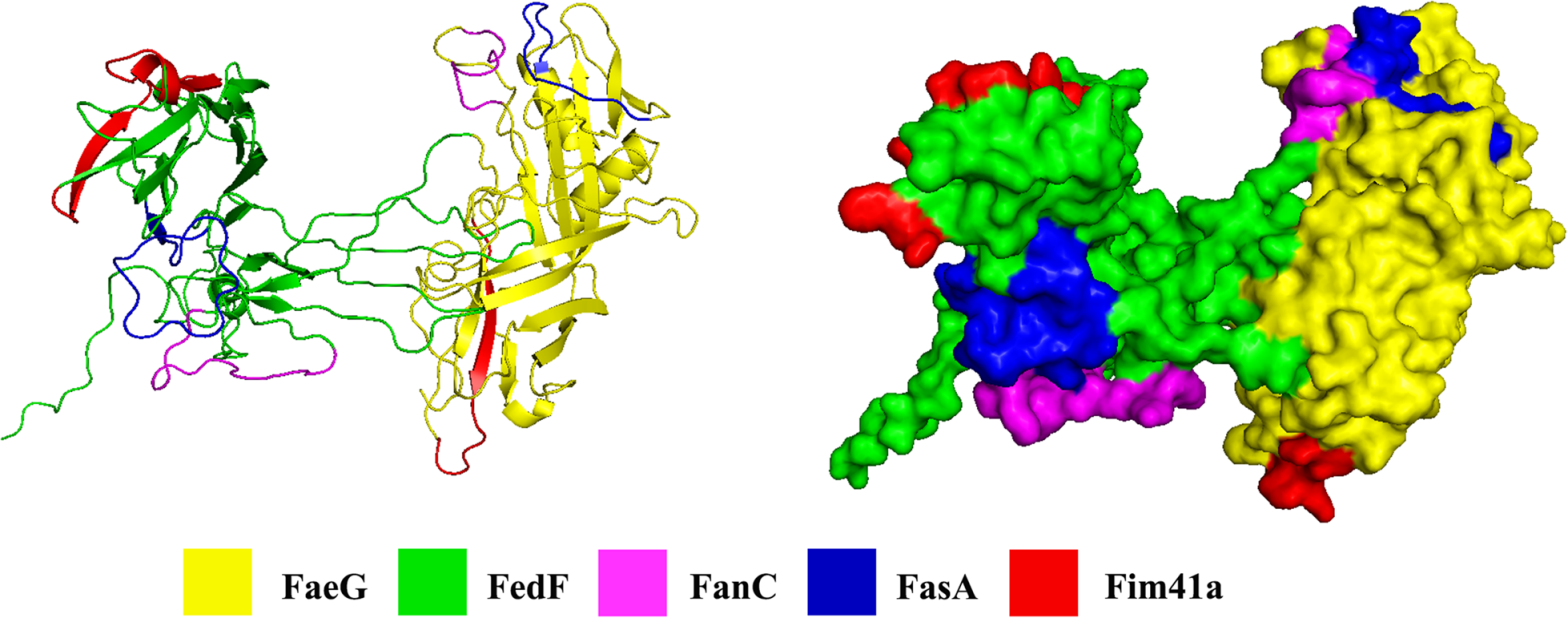
**Computational model of FaeG–FedF–FanC–FasA–Fim41a MEFA protein.** Rosetta was used to construct protein models, using the K88 fimbriae major structural subunit FaeG model (PDB ID: C2j6gA) as the template. Epitopes of the adhesive subunits of the five fimbriae are highlighted in different colors: FaeG (yellow), FedF (green), FanC (pink), FasA (blue), and Fim41a (red).

Three-dimension protein modeling program and PyMOL molecular graphics system were collectively used to generate a predicted structure for the FaeG–FedF–FanC–FasA–Fim41a MEFA protein based on its amino acid sequences. In total, 98 models were generated and the model with the top conformer score was selected as the final model after comparison with all 98 models. Protein modeling indicates that all inserted epitopes of K99, 987P, and F41 fimbriae were displayed on the surface of the FaeG–FedF–FanC–FasA–Fim41a MEFA protein (Figure 2).

### Mice SC co-immunized with FaeG–Fim41a–FanC–FasA and FedF–FasA–Fim41a–FanC MEFA proteins developed antibody responses to each fimbria subunit

**Figure 3:**
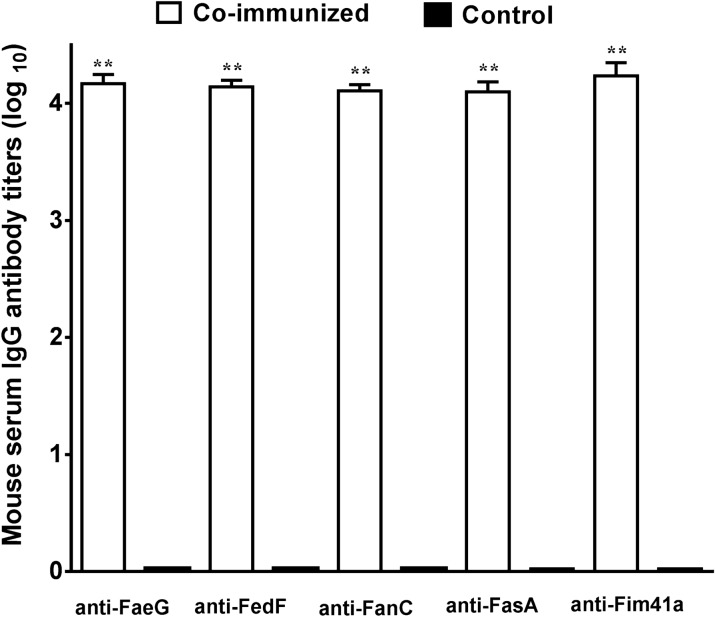
**Mouse serum IgG antibody titers (log**_**10**_**) to FaeG, FedF, FanC, FasA, and Fim41a subunit induced from SC co-administration with FaeG–Fim41a–FanC–FasA and FedF–FasA–Fim41a–FanC MEFA proteins.** Boxes in white are IgG titers from the SC co-immunization group. Boxes in black are IgG titers from the control group. Bars indicate standard deviations of IgG titers from individual mice in the immunized group or the control group. **Statistically significant difference when compared to the control group (P < 0.01).

Mice co-immunized with FaeG–Fim41a–FanC–FasA and FedF–FasA–Fim41a–FanC MEFA recombinant proteins developed antibody responses to the FaeG, FedF, FanC, FasA, and Fim41a fimbrial subunits (Figure 3). Mouse serum anti-FaeG, -FedF, -FanC, -FasA, and anti-Fim41a IgG titers were 4.10 ± 0.08, 4.11 ± 0.05, 4.14 ± 0.06, 4.17 ± 0.08, and 4.23 ± 0.11 (log_10_), respectively in the co-immunized group. No anti-fimbriae subunit IgG antibody responses were detected in the control group serum.

### Mice SC immunized with FaeG–FedF–FanC–FasA–Fim41a MEFA protein developed antibody responses to all five fimbriae adhesive subunits

**Figure 4:**
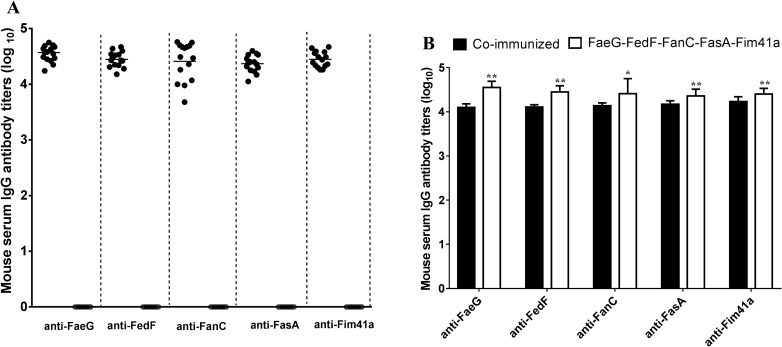
**Mouse serum anti-fimbriae IgG antibody titers (log10) from SC immunized with FaeG–FedF–FanC–FasA–Fim41a MEFA protein. A** Titration of anti-FaeG, FedF, FanC, FasA, and Fim41a IgG antibodies in serum samples of the FaeG–FedF–FanC–FasA–Fim41a MEFA protein immunized mice (filled circle) and control mice (open circle). Each circle represents the IgG titer from a single mouse. Bars indicate the mean titers of the group specific to each fimbria. Antibody titers are presented as log_10_. **B** Comparison of the specific anti-fimbriae antibody titers between the co-administration group and FaeG–FedF–FanC–FasA–Fim41a MEFA group. *Statistically significant difference when compared to the co-administration group (P < 0.05), **Statistically significant difference when compared to the co-administration group (P < 0.01).

Mice immunized with FaeG–FedF–FanC–FasA–Fim41a MEFA protein were expected to develop antibodies against all five fimbriae adhesive subunit proteins present in the fimbriae-targeted MEFA proteins (Figure 4A). Indirect ELISA using recombinant fimbriae subunit proteins as coating antigens detected anti-FaeG, -FedF, -FanC, -FasA, and anti-Fim41a IgG antibody titers at 4.55 ± 0.14, 4.45 ± 0.14, 4.41 ± 0.34, 4.36 ± 0.15, and 4.40 ± 0.13 from the serum samples of the immunized group. No antibody responses to these fimbriae subunit antigens were detected from the serum samples of the control mice. The anti-FaeG, -FedF, -FanC, -FasA, and anti-Fim41a IgG antibody titers in the FaeG–FedF–FanC–FasA–Fim41a MEFA protein were all significantly higher than those in the co-immunized group (Figure 4B).

### Serum samples from immunized mice inhibited adherence to porcine intestinal epithelial cells of ETEC bacteria expressing the corresponding fimbriae

**Figure 5:**
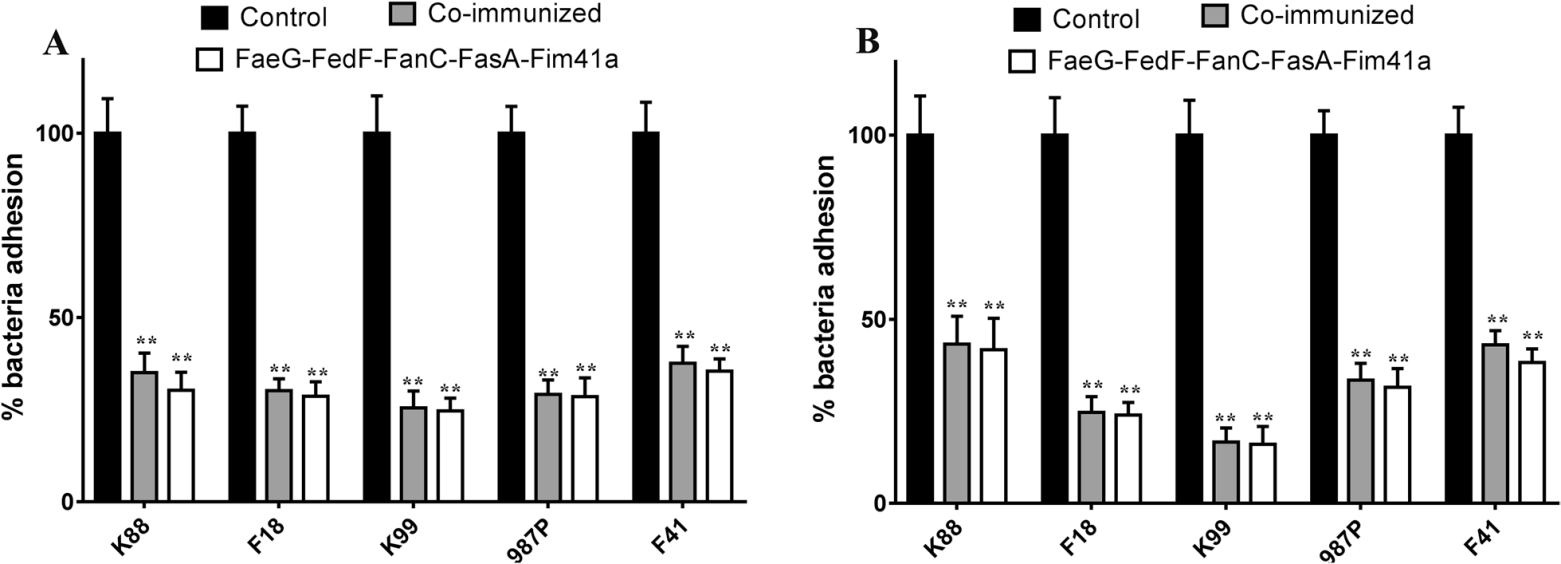
**Anti-fimbriae antibodies from the immunized groups inhibited adherence of porcine ETEC strains to porcine small intestinal cell lines. A** Serum samples from the immunized groups significantly inhibited K88+, F18+, K99+, 987P+, and F41+ ETEC strains to the IPEC-1 cell line. **B** Serum samples from the immunized groups significantly inhibited K88+, F18+, K99+, 987P+, and F41+ ETEC strains to the IPEC-J2 cell line. The number of adherent bacteria (CFU) was converted to percentages, with CFU from cells treated with control group serum set to 100%. The results are expressed as mean ± standard deviation of at least three independent experiments in three wells. **Indicates statistically significant difference when compared to the ETEC strains treated with control serum (P < 0.01).

Serum samples from mice either immunized with FaeG–FedF–FanC–FasA–Fim41a MEFA protein or co-immunized with FaeG–Fim41a–FanC–FasA and FedF–FasA–Fim41a–FanC MEFA proteins show significantly reduced adherence of ETEC bacteria producing K88, F18, K99, 987P, or F14 fimbriae in both the IPEC-1 (Figure 5A) and IPEC-J2 cell lines (Figure 5B) (P < 0.01), when compared to the ETEC strains incubated with the pooled serum from the control mice. Pre-incubation with serum samples obtained from the co-immunized group revealed K88+, F18+, K99+, 987P+, and F14+ ETEC strains had reduced adherence to IPEC-1 cells by approximately 65%, 70%, 75%, 70%, and 63%, respectively; comparatively, strains had reduced adherence to IPEC-J2 cells by 57%, 76%,84%, 67%, and 57%, respectively. These reductions were significant when compared to the same bacteria pre-incubated with serum samples from the control group. Likewise, pre-incubation with serum samples obtained from the FaeG–FedF–FanC–FasA–Fim41a immunized group revealed adherence reductions to IPEC-1 cells in the K88+, F18+, K99+, 987P+, and F14+ ETEC strains by approximately 70%, 71%, 75%, 72%, and 65%, respectively; comparatively, strains had reduced adherence to IPEC-J2 cells by 58%, 76%, 84%, 69%, and 62%, respectively. These reductions were significant when compared to the same bacteria pre-incubated with serum samples from the control group. However, serum samples from these two immunized groups had no significant differences in adherence inhibition to either IPEC-1 or IPEC-J2 cells from all five ETEC strains.

## Discussion

PWD caused by ETEC remains a serious problem in newly weaned piglets, resulting in considerable morbidity, mortality, and enormous global economic losses in swine husbandry [1]. In general, neonatal diarrhea in piglets can be effectively protected against by passive colostral and lactogenic immunity obtained from vaccinated sows; however, current measures have not shown effectiveness against ETEC-driven PWD in pigs [15]. To date, vaccination has been considered an effective and probably the most sustainable strategy to prevent against ETEC-associated PWD [1, 23]. The mechanism by which the vaccine works is based on its ability to block fimbria-mediated initial ETEC adhesion to the pig’s enterocytes. Moreover, epidemiologic studies have shown that both K88 and F18 fimbriae are more prevalent than other fimbriae in porcine ETEC strains associated with PWD [4, 5]. Fimbriae are also thought to be very good particle immunogens because they are proteinaceous and contain a set of epitopes that are repeated 10^2^ to 10^3^ times on each fimbrial thread [24]. Based on this concept, various active immunization studies have focused on the relevant K88 and F18 fimbriae antigens, including purified fimbriae, recombinant fimbrial adhesive subunits, or fimbriae-positive non-pathogenic *E. coli* to protect piglets against PWD caused by K88+ and/or F18+ ETEC infections. Recently, it has been demonstrated that two live vaccines (Coliprotec^®^F4 and Coliprotec^®^F4/F18) developed by Elanco protected weaned piglets from PWD caused by F4+ and F18+ ETEC [20, 25]. Importantly, oral administration of a single dose of Coliprotec^®^F4 in newly weaned piglets at least 18 days of age protected them against an F4+ ETEC challenge until 21 days post vaccination [25]. Meanwhile, oral immunization of recently weaned piglets (≥ 18 days) with one dose of live, bivalent Coliprotec^®^F4/F18 protected piglets who were then challenged within 7 days of vaccination with both F4+ ETEC and F18+ ETEC. Protection against F18+ ETEC lasted for at least 21-days post-vaccination [20]. Despite this promising past work, current vaccines have failed to provide cross-protection against PWD infected by the ETEC expressing K99, 987P, and F41 fimbriae. This lack of cross-protection is because there is no cross-reactivity between K88, K99, 987P, F18, and F41 fimbriae. Though the predominant fimbrial types of ETEC isolated associated with cases of PWD are K88 and F18 fimbriae, K99, 987P, and F41 fimbriae have also been detected with different rates in different regions [4, 6, 7]. Therefore, each of these fimbria types needs to be included in the vaccine to allow for the broadest level of protection.

Unfortunately, developing better vaccines against ETEC bacterial adherence and colonization based on this targeted fimbriae approach remains difficult. The major challenge to this method is the genetically and immunologically heterogeneous fimbriae that are expressed by porcine ETEC strains [26]. Those involved in PWD include five heterogeneous fimbriae virulence factors. Moreover, the immune response induced by one of the fimbria types provides protection only against ETEC strains expressing the same type of fimbria and is unable to provide cross-protection against other ETEC strains expressing a different fimbria type.

To overcome these aforementioned challenges, an alternative approach to vaccine development that encompasses all the adhesive subunits of the five fimbria types is urgently needed. MEFA is a structure-based vaccine development technology that combines the power of computational biology with structural biology to create multivalent vaccines that incorporate the expected protective epitopes of heterogeneous virulence factors into a single protein [27, 28]. In this study, the single fimbriae-targeted MEFA platform incorporated the expected protective epitopes from five different types of fimbriae expressed by PWD-causing ETEC strains. This MEFA protein was then created and—after immunization with it,—elicited antibodies against each fimbria included. In vitro work also shows that it effectively neutralizes ETEC attachment to intestinal epithelial cells. Importantly, this is the first report of a fimbriae-based multivalent vaccine that is capable of inducing antibodies against all five types of ETEC fimbriae associated with PWD.

Data from our murine experiments indicate that either co-administration of FaeG–Fim41a–FanC–FasA and FedF–FasA–Fim41a–FanC MEFA proteins or mono-administration with FaeG–FedF–FanC–FasA–Fim41a MEFA protein was capable of inducing a strong immune response to each of the five ETEC fimbriae. It is worth noting that the anti-fimbriae antibody titers corresponding to each fimbria in the group immunized with only the FaeG–FedF–FanC–FasA–Fim41a MEFA protein were significantly higher than those in the co-immunized group. A previous study indicated that a MEFA toxoid carrying three copies of the STa toxoid induced higher anti-STa antibody titers than those induced by the one carrying only one copy [29]. To obtain the expected higher levels of immune responses to each fimbria, we used both FaeG and FedF subunits as the backbone, each of which carried one epitope for the FanC, FasA, and Fim41a subunits. Finally, two epitopes of each fimbria subunit were carried by the FaeG–FedF–FanC–FasA–Fim41a MEFA protein. Therefore, the higher antibody titers in the FaeG–FedF–FanC–FasA–Fim41a MEFA group were attributable to the MEFA presenting and displaying more epitopes at the same dose. As ETEC infections are non-invasive gastrointestinal infections, protection efficacy may depend on local mucosal immunity with anti-fimbria-specific secretory IgA in the local environment of the small intestine [15]. Since the oral route is the most preferable delivery method to obtain an active intestinal mucosal immune response, delivery of the fimbria MEFA protein by a virulent host strain and subsequent induction of small intestinal mucosal immunity is an important next step to optimize future vaccine formats.

Previous studies have shown that both a CFA/I/II/IV MEFA carrying epitopes of the major subunits of the seven most important human ETEC adhesins [30] and another type of CFA adhesin tip MEFA [22] that carried the epitopes of the tip subunit of nine important human ETEC adhesins were able to induce protective antibodies against adherence of *E. coli* strains to Caco-2 cells expressing the corresponding adhesin. Similarly, our data indicate that antibodies derived from both immunized groups significantly inhibited adherence of porcine ETEC bacteria expressing K88, F18, K99, 987P, and F41 fimbriae to both IPEC-1 and IPEC-J2 cell lines. These cell lines are derived from either the small intestine or jejunum of neonatal piglet and used as in vitro cell models to investigate the interaction between porcine ETEC strains and porcine intestinal epithelial cells. Our results show that a MEFA protein carrying one or two epitopes of the adhesive subunit of the included fimbriae was sufficient to induce neutralizing anti-fimbriae antibodies. These results will completely change the current view that induction of protective anti-fimbriae antibodies requires either the entirety of the fimbria antigen or the adhesive subunit of the fimbria. Moreover, our results provide additional evidence that MEFA is a promising technology for the development of effective, multivalent vaccines that are broadly protective against heterogeneous pathogens and virulence factors. This work may also rejuvenate the concept of epitope vaccines, in that multiple neutralizing epitopes from heterogeneous pathogens and/or virulence factors can be integrated into a single immunogen; importantly, this approach mimics epitope native antigenicity, forming a multivalent antigen to induce broadly protective immunity.

## Conclusion

Our results indicate that in mice, either SC co-administered with FaeG–Fim41a–FanC–FasA and FedF–FasA–Fim41a–FanC MEFA proteins, or immunization with FaeG–FedF–FanC–FasA–Fim41a MEFA protein alone was capable of inducing production of antibodies that cross-reacted with K88, K99, 987P, F18, and F41 fimbriae. More importantly, the derived, specific anti-fimbriae antibodies significantly inhibited adherence of porcine ETEC bacteria expressing these five fimbriae to porcine small intestinal epithelial cells. These findings indicate that the fimbriae-targeted MEFA is a promising candidate for the production of an efficient PWD vaccine. ETEC strains expressing K88, K99, 987P, F18, and F41 fimbriae are also an important bacterial cause of neonatal diarrhea in piglets; therefore, these fimbriae MEFA are promising vaccination approaches for sows to induce lactogenic immunity. This is another means by which piglets could be protected from neonatal diarrhea caused by ETEC. Future work will investigate the optimized vaccine format to elicit both systemic and mucosal immune responses, coupled with pig challenge studies to verify the fimbriae-targeted MEFA-induced protective efficacy. This future work will allow a better evaluation regarding the use of ETEC vaccine candidates against both PWD and neonatal diarrhea in piglets.

## Data Availability

The datasets analyzed during the current study are available from the corresponding author on reasonable request.

## Abbreviations

AMR: antimicrobial resistance
B-PER: bacterial protein extraction reagent
CIVDC: China Institute of Veterinary Drug Control
CFUs: colony-Forming Units
DMEM: Dulbecco minimal Eagle medium
ETEC: enterotoxigenic *Escherichia coli*
FBS: fetal bovine serum
IPTG: isopropyl-β-d-1-thiogalactoside
LT: heat-labile enterotoxin
MEFA: multi-epitope fusion antigen
PBS: phosphate buffer saline
PWD: post-weaning diarrhea
SC: subcutaneously
SDS-PAGE: sodium dodecyl sulfate polyacrylamide gel electrophoresis
SOE PCR: splicing overlap extension PCR
ST: heat-stable enterotoxin
TMB: 3, 3′, 5, 5′-tetramethylbenzidine
